# Understanding the Academic Success of Academically Talented College Students with Autism Spectrum Disorders

**DOI:** 10.1007/s10803-021-05290-4

**Published:** 2021-10-21

**Authors:** Sally M. Reis, Nicholas W. Gelbar, Joseph W. Madaus

**Affiliations:** Department of Educational Psychology, Neag School of Education, University of Connecticut, 2131 Hillside Road, Unit 3007, Storrs, Mansfield, CT 06269-3064 USA

**Keywords:** Autism, Twice-exceptional, Academically talented, High school academic success, Transition to college

## Abstract

Little is known about the academic and extra-curricular experiences of academically talented students with Autism Spectrum Disorder (ASD). This study focused on how these capable students with ASD successfully navigated and completed high school, and specifically, the experiences that enabled them to attend competitive colleges. Using comparative case studies and directed content analysis, data were derived from semi-structured interviews with 40 students who had been identified as academically talented with ASD, and were enrolled in, or recently graduated from, highly competitive colleges in the United States. The majority were identified as having academic talents, participated in challenging honors classes, enrichment opportunities, interest-based extra-curricular activities, residential summer programs, and pursued other advanced educational experiences. Implications for educational and talent development services are included.

Academically talented high school students with Autism Spectrum Disorder (ASD; Assouline et al., 2012) are often labeled as twice-exceptional (2e). Many parents and educators do not understand how to support these intelligent young people with ASD to help them successfully complete high school and attend competitive colleges. This research investigated the academic and extra-curricular and related experiences that enabled 40 students who were both academically talented and identified with ASD to successfully navigate high school and matriculate in competitive colleges and universities. It probed participants’ perceptions about their academic work, as well as the extra-curricular experiences, study, and social strategies that enabled them to succeed in high school. Data were derived from semi-structured interviews about students’ academic experiences, extra-curricular activities, learning and compensation strategies, and social interactions in high school.

## Defining Twice-Exceptional Students

The term, 2e, is utilized to describe students who have *both* gifts/talents and disabilities. Over three decades of scholarship has indicated that the needs of this group overlap and yet are distinct from both individuals who are gifted and individuals with disabilities. The 2e population represents the intersection of gifts/talents and disabilities and as such, multiple definitions for this population have been proposed in the literature, with varying definitions of giftedness and disabilities.

Many broad conceptions of giftedness exist (Sternberg & Ambrose, 2021; Sternberg & Davidson, 1986, 2005), each influenced by the culture, environment, and context in which gifts emerge and are developed (Renzulli & Reis, 2021). Young people identified as gifted are a diverse group exhibiting a wide range of characteristics in ability and achievement, temperament, and effort invested in reaching goals. A summary of the research on gifted and talented learners demonstrates that existing definitions of giftedness fall into various broad categories, including those based on high aptitude and IQ, exceptional abilities and talents, high creative productivity, and/or high level performance across domains or varied fields of activities (Sternberg & Ambrose, 2021; Sternberg & Davidson, 1986, 2005; Subotnik et al., 2012). Renzulli has made important distinctions between giftedness based on aptitude and intelligence tests and creative productive giftedness, and is often cited in the research for his three ring, broadened conception of giftedness (Renzulli, 1978; Renzulli & Reis, 2021). Accordingly, this conception can be extremely helpful in considering the talents of students who are 2e.

The 2009 Joint Commission on Twice-Exceptional Students developed the following widely accepted definition of 2e, which is used as the operational definition of 2e students used for this study:

Twice-exceptional learners are students who demonstrate the potential for high achievement or creative productivity in one or more domains such as math, science, technology, the social arts, the visual, spatial, or performing arts or other areas of human productivity AND who manifest one or more disabilities as defined by federal or state eligibility criteria (Reis et al., 2014).

In the United States, students who are twice-exceptional are increasingly being identified in schools and have been the focus of new research, but a clear consensus does not exist about defining this population and scholars agree that more data-based research needs to be conducted (Baum et al., 2014; Foley Nicpon et al., 2011, 2012). For example, educators know little about how many 2e students are actually identified and served in the United States (Reis et al., 2014). Young people identified as 2e rarely have both exceptionalities identified while they are in elementary or secondary school (Reis et al., 1997). In some cases, their advanced abilities are identified first, but most often their disabilities are identified, while their academic talents are not (Reis et al., 2014). When this occurs, 2e students’ academic and social needs may not be met and ultimately they may not receive appropriate clinical and educational intervention services (Assouline et al., 2009).

Much of the initial work on 2e students focused on students with learning disabilities (e.g., Vaughn, 1989), but over time, a broader pool of gifted and talented students was identified as having a range of disabilities, including Attention Deficit Hyperactivity Disorder (ADHD; e.g., Webb & Latimer, 1993), and Autism Spectrum Disorder (e.g., Barber, 1996). Researchers who conduct research about 2e students regularly discuss the absence of empirical research that identifies and delineates the unique needs of this population and how to support their educational needs (Reis et al., 1997; Foley Nicpon et al., 2011). Other concerns with the current state of the 2e research include a limited literature base with: (a) small sample sizes (Assouline & Whiteman, 2011; Wang & Neihart, 2014; Wu et al., 2019); (b) multiple exceptionalities present in the same study (Reis et al.,  1997; Willard-Holt et al., 2013); (c) inclusion of the perspective of teachers or parents, but not of students (Neumeister et al., 2013; Schultz, 2012); and (d) treating 2e students as homogenous while in fact there is distinct intra-group variation (Reis et al., 1997) due to both the types of gifts/talents and disabilities present in individuals. One recent study illustrates the type of recent research that has been conducted. Using a success case methodology, Wu et al. (2019) completed an in-depth analysis of two fifth graders who were gifted with ASD, finding benefits of a supportive and safe school context, implementing curriculum flexibility, and a strength-based approach.

The extant research findings that guided our directed content approach identified the following: (a) 2e students often receive services focusing on remedial intervention (Reis et al., 1997; Crim et al., 2008); (b) effective teaching for 2e students should focus on finding a balance between fostering students’ academic strengths and interests and helping them learn to overcome their learning difficulties (Baum et al., 2014; Foley Nicpon et al., 2011; Mann, 2006; Wang & Neihart, 2014); (c) the use of enrichment and strength-based strategies to enhance learning and healthy social and emotional development (Reis et al., 1997; Neumeister et al., 2013); and (d) extra-curricular activities seemed beneficial for some 2e college students (Reis et al., 1997). To illustrate one finding, Crim and colleagues (2008) studied the Individualized Education Programs (IEPs) of 1055 students receiving services for specific learning disabilities, and found that of this group, 112 were identified as high-ability students because their FSIQ was 116 or greater. Of this group, not one student was nominated for gifted and talented services or recommended to receive any advanced or differentiated type of educational modifications that addressed their abilities or talents. It is important to note that these students were identified with learning disabilities and no comparable research has been conducted about the IEPs of individuals with ADHD or ASD.

### Defining 2e/ASD

Less scholarship has focused on academically talented students with ASD (Gelbar et al., in press). ASD is a complex, chronic neurodevelopmental condition, clinically characterized by significant impairment in core behavioral domains including social interaction and communication and restricted repetitive behaviors and/or interests (APA, 2013). A recent systematic review focused on academically talented students with ASD (2e/ASD; Gelbar et al., in press) found that 32 articles had been published on the topic prior to December 31, 2019. Twenty of these articles presented data, and the preponderance of the scholarship established that there are individuals with ASD with academic talents. Twice-exceptional students with ASD may exhibit certain common characteristics or features, such as difficulties with social interaction, emotional maladjustment, pedantic use of language, excessive focus on special interests, specific sensorial characteristics, or attention deficits (Doobay et al., 2014).

One of the challenges of defining individuals who are 2e/ASD is that it may be difficult to differentiate ASD from advanced abilities or giftedness (Assouline et al., 2012). In other words, high ability and academically talented children who are advanced in school may find it challenging to interact with same age peers, which can make it difficult to determine if they have the hallmark social deficits of ASD or are just more advanced than their peers. Further, much of the research concerning individuals with ASD has focused on remediating their difficulties as opposed to developing their strengths (Gelbar et al., in press), again pointing to the need for new data-based research. In most research conducted on 2e/ASD, the students’ ASD remains the primary focus of their educational experiences (Gelbar et al., in press). No research was found that investigated evidence-based best practices for supporting 2e/ASD students in high school when they are enrolled in challenging courses. Little research has been conducted about the academic experiences necessary for their academic success in high school and beyond (Anderson et al., 2018; Gelbar et al., 2015; Shattuck et al., 2012). Little research delineates the unique needs of this population and how best to support their education (Foley Nicpon et al., 2011).

As the number of students with ASD who receive special education services nationally has increased from 1.5% of the total percentage of students in 2000–2001 to 9% in 2015–2016 (NCES, 2019), this research is critical. Data about percentages of academically talented students with ASD who attend and either leave or graduate from college are difficult to obtain, but some trends are available. For example, data from the National Longitudinal Transition Study-2 (NLTS-2) indicate that 34.7% of youth with ASD transitioned to postsecondary education, with most (28%) attending 2-year colleges (Shattuck et al., 2012).

In summary, little research exists about students who are 2e/ASD and their high school experiences, including how many are identified as having academic talents, how many receive advanced learning opportunities in high school, the types of academic and extra-curricular experiences that help them succeed academically in high school, the types of learning, study, and interpersonal strategies that enable them to succeed academically, and other high school experiences that contribute to their academic success. This absence of research knowledge can result in serious challenges for educators, parents, and policy makers who would benefit from understanding which educational interventions enable 2e/ASD students to succeed in school (Foley Nicpon et al., 2011). The research described in this article is important as a majority of 2e students, across various exceptionalities, believe that their school experiences failed to facilitate the development of their full potential (Willard-Holt et al., 2013). As this review of the literature corpus suggests, a paucity of research evidence exists about what works for 2e/ASD students who are academically successful, complete high school, and attend competitive colleges (Assouline et al., 2012).

### Study Purpose

The purpose of this study was to investigate the intellectual, learning, educational, and social characteristics and experiences of academically talented youth with an ASD diagnosis, utilizing interviews with 40 participants. This is an important area for researchers, educators, and parents, as increased awareness and deeper understanding is needed about how 2e/ASD students succeed in school. As noted, many students with talents and disabilities are not successful in college, wasting the resources and talents of these young people and their families and leading to frustration, anxiety, depression, early withdrawal, and loss of opportunities (Reis et al., 1997; VanBergeijk et al., 2008). Educators and researchers should also note that college may not be the only path to success for this groups of students, as some may want to pursue more technical careers or other opportunities for future careers and post-secondary education. College graduation is only one measure of success for both this group, and indeed, other students as well. If students and their parents do want to pursue college, however, they should understand how high potential students can be challenged in high school to prepare them to succeed academically and socially and attend colleges of their choice, including competitive institutions. Educators and parents would also benefit from understanding the types of academic and extracurricular activities that successful 2e/ASD students believe contributed to their academic achievement and ability to matriculate in competitive colleges. The following research questions guided this study:

1. What are the demographic and related characteristics of academically talented students with ASD who were successful in high school and are attending or recently graduated from competitive colleges?
2. What are the academic, social, and extracurricular experiences of academically talented students with ASD, who were academically successful in high school, that facilitated their ability to attend competitive colleges?

## Research Methods

Semi-structured interviews were conducted with 40 college students with ASD to gather data to answer the research questions that guided this study. Each researcher conducted between 10 and 15 interviews. The present study contributes to minimal existing research about the educational and academic experiences of academically successful 2e/ASD students.

### Methodology

Multiple case study research was used in this study to analyze and synthesize similarities, differences, and patterns across multiple cases (Baxter & Jack, 2008; Flyvbjerg, 2013; Goodrick, 2014; Stake, 2006). Examining a common focus across multiple cases, in this case, the educational and social high school experiences of academically successful 2e/ASD students, enabled researchers to analyze how and why particular situations succeeded or failed for this population in high school. This design integrates both qualitative and quantitative methods, which are useful for understanding the participants, the context of their school-based programs, and how programs can be implemented or changed to achieve the intended outcomes. A multiple case study approach (Baxter & Jack, 2008; Flyvbjerg, 2013; Stake, 2006) enabled researchers to gain an understanding of students’ perceptions of their high school experiences, facilitating the investigation of multiple high school experiences that contributed to their success. Our goal for this study was to describe this population and the positive high school experiences that enabled them to be accepted at and attend colleges, using qualitative description as our primary mode of inquiry (Lambert & Lambert, 2012; Sandelowski, 2000).

Sample size guidelines suggested a range between 20 and 30 interviews would be adequate (Creswell, 2007), but we continued until 40 participants had been interviewed, using purposive sampling to select ‘information-rich’ cases. Researchers critically considered saturation parameters found in prior methodological studies and applied them to this study to determine when data adequacy and saturation were reached (Ritchie et al., 2003; Vasileiou et al., 2018). Methods included having all three researchers code transcribed interview data, as well as constructing a data grid of participants, during and after the period in which interviews were being conducted (Brod et al., 2009).

### Recruitment of Participants and Interview Procedure

After this study was approved by the university IRB, recruitment e-mails were sent to administrators of disability service offices at five competitive colleges who were asked to forward recruitment information to students with ASD. In addition, alumni from an independent school that provides support to students who are 2e were sent recruitment information.

Interested students were asked to confirm that they met the study’s inclusion criteria. To be included in the study, each participant: (a) was currently in college or had recently completed college; (b) had a previous ASD diagnosis from an educational setting, clinical psychologist, or psychiatrist; (c) was identified as ASD, had disclosed the disability, and was receiving services from a university or college center for students with disabilities; (d) had school-based evidence of academic talents and high potential, including being formally identified as gifted or talented in school, participation in gifted education programs or services, and participation in accelerated and advanced classes, if a formal gifted education program was not available in their high school; and (e) agreed to participate in the interviews and any follow-up questions that emerged. If they met all of the criteria, their informed consent was obtained, and participants were given a $20.00 gift card for their participation in the study.

After receiving their informed consent, participants were asked their preference about completing an in-person interview or a telephone interview (preferred by many due to the COVID virus quarantine), that included several demographic questions related to their high school and college experiences, including grades, test scores, activities, as well as their academic and extracurricular activities, with several open-ended questions relating to various experiences that were most important to their academic success. The questions were constructed by the investigators and were reviewed and field tested by the researchers to reach consensus regarding final wording and ordering. The questions were administered identically by all three researchers who conducted the interviews. All interviews were transcribed to written text for analysis. Interviews were approximately an hour in length and follow-up interviews were conducted and additional follow-up questionnaires were sent for clarification of responses after the transcripts were reviewed by all researchers, to which 31 participants responded. Member checks were also conducted, as each participant was given the opportunity to review their transcript for accuracy. Each participant was asked to review his/her transcript and email any edits or additional comments or corrections. Three participants responded with comments acknowledging the accuracy of the transcript and offered no substantive edits to the transcripts.

### Data Analysis

Directed content analysis is often used to both describe and quantify phenomena (Elo & Kyngäs, 2008), as some codes were previously determined in sparse but important earlier research (Reis et al., 1997; Baum et al., 2014; Foley Nicpon et al., 2011; Wang & Neihart, 2014) and others emerged during data analysis (Hsieh & Shannon, 2005). This process was used in this study to enable researchers to both investigate these previous research findings as well as identify new findings. This method enabled researchers to both inductively code participant interview data using research-based codes (e.g., strength-based academic experiences) and identify new codes from new data (e.g., the importance of leadership in extracurricular activities). All researchers participated in analyzing data for this study, independently coding responses, and debriefing to discuss emerging findings based on previous research as well as identifying new codes and findings (Krefting, 1990). A thorough qualitative analysis of the transcript was conducted by the researchers and preliminary themes were identified. All researchers then agreed upon a consensus for a code book, including shared definitions of each code. The authors then conducted a thematic analysis using procedures suggestions by Braun and Clarke (2006). The codes were re-read and integrated into broad themes and categories, and then reviewed again. Categories that did not have enough data to support identification as themes were collapsed and preliminary themes were developed by all three researchers. We made extensive use of the code book, spread sheets, and tables to review the coded data extracts for each theme to consider whether they appeared to form a coherent pattern. The validity of each theme was considered to determine whether the themes accurately reflect the meanings evident in the data set as a whole, using suggestions from Braun and Clarke (2006). Data were recoded multiple times, enabling the identification of the themes that had the most substantial data and those that did not have enough data to support them.

Although minimal research has been conducted on the high school experiences of academically talented students with ASD, some researchers have identified factors related to successful academic and social experiences that may have positively affected a broader pool of 2e students’ positive educational achievement. These factors, as reviewed in this article, include finding a balance between fostering students’ academic strengths and interests and helping them learn to overcome their learning difficulties (Reis et al., 2014; Baum et al., 2014; Foley Nicpon et al., 2011; Mann, 2006; Wang & Neihart, 2014); and the use of enrichment and strength-based strategies provided by educators, and parents to enhance learning and healthy social and emotional development (Baum et al., 2014; Neumeister et al., 2013).

Before coding, the researchers independently read over 300 pages of participant interview transcripts several times to gain a general understanding of the data. The researchers first individually coded responses to questions that probed high school academic experiences and categorized responses. Transcripts were also analyzed and assessed for new and emerging themes. The researchers independently, and then collectively, inductively coded for new themes that represented a different high school success factor, and when identified, coded these as a new theme. Once the initial coding scheme was completed, the selected new codes were discussed and evaluated by the research team, as a group, and a final consensus was reached across codes (Potter & Levine-Donnerstien, 1999).

### Trustworthiness and Credibility

Various strategies were used to establish trustworthiness that included credibility, transferability, dependability, and confirmability. Member checks were used to verify interview data with participants (Anney, 2015; Baxter & Jack, 2008; Stake, 2006). The data from the interviews was used to develop convergent evidence to support findings, in turn strengthening the validity or credibility (Merriam & Tisdell, 2016). Triangulation was also used in the cross-case analysis, ensuring that findings from different cases support final assertions (Merriam & Tisdell, 2016; Stake, 2006). Samples of data, both across cases and within identified themes, are provided in this article to illustrate details in the case narratives and verify coding to ensure that the validity and credibility of the work can be established (Baxter & Jack, 2008). Triangulation and the inclusion of all research team members in the coding process supported dependability, as did an audit trail to establish the dependability of this research (Anney, 2015; Merriam & Tisdell, 2016). Using a multi-case approach supported the transferability of the findings due to the richness of multiple cases (Merriam & Tisdell, 2016). In summary, an audit trail, data tables and matrices, member checks, and all senior researchers’ involvement in coding data contributed to confirmability (Amankwaa, 2016; Anney, 2015).

### Participants

Of all participants, 39 (98%) were formally diagnosed with ASD using contemporary guidelines, which means that this group includes individuals who were initially diagnosed with Asperger’s Syndrome or Pervasive Developmental Disorder-Not Otherwise Specified. One was initially diagnosed with Social Communication Disorder and an unspecified personality disorder, and later self-identified as having ASD. Most could explain exactly when and how they were diagnosed as having ASD. As participants were recruited primarily from competitive colleges with centers for students with disabilities that provided services, it should also be noted that extensive evidence was required, including current assessments for eligibility for services. All study participants qualified for services from one of these centers. All participants had been identified as having academic talents, usually by aptitude test scores, participation in gifted and enrichment programs, and school-based advanced program and class participation.

All participants were either enrolled in or recently graduated from competitive or very competitive colleges and universities, according to Barron’s classification. As indicated in Table 1, their responses indicated that 23% were freshmen, 13% sophomores, 20% juniors, 15% seniors, and 10% had recently graduated and were in graduate school. The remaining participants explained that it was difficult to categorize their year in college, as they were between two years, as Georgia explained, “I am in my fourth year, but credit wise I’m still a junior, so next year will be senior year. So I’ll graduate in 5 years. I took an extra year as I didn’t have enough credits.”

**Table 1:** Descriptive characteristics of participants (*n* = 40)

Demographic (range)	*n*	%
Gender		
Female	9	22.5%
Male	27	67.5%
Other (non-binary and/or transgender)	4	10.0%
Educational level (year in college)		
Freshman	9	22.5%
Sophomore	5	12.5%
Junior	8	20.0%
Senior	6	15.0%
Difficult to categorize (i.e., between two years)	8	20.0%
Graduate student	4	10.0%
High school GPA (2.0–4.0)		
Earning a GPA between 3.5 and 4.0	25	62.5%
Achieved a GPA between 3.0 and 3.4	8	20.0%
Identified as gifted or academically talented	21	52.5%
Participation in		
Extracurricular activities—Sports/clubs	36	90.0%
Advanced/accelerated courses	29	72.5%
Residential programs	20	50.0%

The participants were primarily male, with 27 (68%) stating they identified as male, nine (23%) indicating they identified as female, and four (10%) stating they identified as non-binary or transgender. The majority were white, and seven participants (18%) indicated that they were culturally and linguistically diverse. Seventeen participants (43%) indicated that they attended a community college or another college before transferring to their current/final college. Most participants (73%) reported that they had already graduated, were graduating the academic year in which the study was conducted, or expected to graduate from college on time, another indicator of their academic success. Most of the participants also earned high GPAs in high school, with 25 (62%) earning a GPA between 3.5 and 4.0. Another 8 (20%) of the participants reported achieving a high school GPA between 3.0 and 3.4. The majority reported attending a public high school, and a quarter of the participants explained that they attended an independent or special education school focused on special needs, 2e, or gifted students. Some participants who attended public schools went to a smaller charter, special education or magnet school within their larger district. One participant took the GED in middle school and was home schooled and attended community college for credit during high school. Students’ responses suggest that those who attended smaller schools, either public or independent, believed that they had more personalized academic support and experiences. One participant, Darrel, noted, “I didn’t go to a public school, but a special needs non-public school that operates differently but still needs to adhere to the code of ethics from the district and board of ed.” Asher described his time at a private school for 2e students as “the best years of my life.”

The majority of our participants reported their scores on the SAT test in high school, and most completed the SAT test when 1600 was the highest score. Others took the ACT. Their self-reported scores on the SAT ranged from 1200 to 1500, with a mean of 1328, and a standard deviation of 92. Participants reported a broad range of scores on the ACT from 18 to 32, with a mean of 26, with a standard deviation of 3.8. One reason for the slightly lower ACT scores might be the variability in content scores. For example, Asher explained, “On the ACT, I did very well—I scored a 25. I had a straight 30–34 on all the other content classes, but I had a 10 in math so that brought my score down.”

In summary, our participant group was primarily male and had very good grades in high school, as well as strong test scores. All participants were either enrolled in or recently graduated from competitive or very competitive colleges and universities The majority of students were still in college, with 4 (10%) having recently graduated, and most participants (73%) explaining that they expected to graduate from college on time.

## Findings

The findings in this study enhance and contribute to research focusing on 2e/ASD students. In addition to the demographic information reported in the description of the participants, other interesting results emerged related to their identification as 2e and their academic and social experiences.

### Identification as 2e (Academically Talented and ASD)

More than half (*n* = 21; 53%) of our participants, indicated that they had been identified as being academically talented in elementary or secondary school, with several suggesting that the tests they had taken to identify their ASD also revealed a pattern of their academic talents and potential. Asher’s explanation was representative of some of the educational experiences of others in this group when asked if they had been identified as gifted or academically talented:

Yes, I don’t like to brag but one of my earliest memories—I was told there was something special about me. I was fixated by fans and solar panels. I had intense interests in strange things—history of logos, the Titanic. My dad first bought me a Smithsonian book on American history when I was very little and I read it cover to cover. I had an amazing appreciation for history and the analytical ability to appreciate history. One year after middle school, American history became a passion. I always enter in calendars when important things happen in peoples’ lives and historical events. I enter stuff in calendars about my family, my girlfriend and then I started entering historical events. I did have a series of tests due to my Asperger’s diagnosis. I also had occupational and speech therapy but my parents and teachers always knew that I was smart.

Adam had a shorter response, stating simply, “Yes, they skipped me a grade and put me in gifted classes.” Avery concurred, explaining, “Yes, they had me in the gifted program. Then after that I went to a gifted school.”

Rachel explained, “Yes. I went to a charter school for (gifted students) in elementary and middle. In first grade I did a lot of 2nd grade work. If I was bored in class, they would give me higher grade level work to do.”

About a quarter of the participants experienced challenges with this dual identification. Matthew discussed some of the challenges he faced as being identified as academically talented, as well as  

I was always told that I was smart…Being smart and on the spectrum is hard. Parents of kids who are both don’t really get the help that they need. Parents of smart kids with ASD too often tell them to do things on their own.

Their identification as both academically talented and having ASD also resulted in some of these 2e students or their parents questioning their diagnosis of ASD and also some of them faced difficulties in school. Molly explained,

My mother was told that I was too smart to have a disability, so I did not really believe that I had a disability. I remember when I was little, in 6th grade and I wrote a story about it—I was kind of a nerd. I listened to Khan Academy in high school and did well on stuff that I liked.

Slightly less than half of the participants (*n* = 19; 47%) explained they were not formally identified or recognized as gifted, some believed mistakenly, as Caitlyn stated, “No, I should have been.” Some of those who were not formally identified attended independent schools that did not identify gifted students, but all who stated that they were not formally identified reported that they had specific advanced, accelerated, or enriched educational experiences. As Chris explained, he had extra credits from a few Advanced Placement (AP) tests, even one in which he did not take the AP history course, and added, “No, but I was a good student and had some enrichment opportunities.”

In some cases, the 2e diagnosis hindered their participation in these classes, potentially due to factors other than advanced abilities. As Jackson explained that he was not identified as gifted and was recommended for advanced opportunities, but he did not always receive them. Jackson elaborated “…the counselors told me that the advanced classes would stress me out—there was a lot going on at home that contributed to my stress.”

In summary, the majority of these participants’ academic talents were formally recognized, as over half reported that they had been identified as being academically talented in elementary or secondary school. Of the remaining group, who were not formally identified, teachers were usually first to recognize their advanced academic talents.

### Advanced Classes and Academic Work

The majority of the participants (*n* = 29; 73%), were enrolled in advanced and accelerated classes, with most reporting taking AP and honors classes in their high schools. Some students completed concurrent enrollment, participating in community college or regular college classes simultaneously during high school. Some who indicated that they did not take honors or advanced classes attended small independent high schools that did not offer these opportunities.

Levi’s response was typical of those who had participated in these classes, stating, “Yes. I took almost all honors, and AP for history and English for American Literature.” Mary explained, “…I did some community college courses and I was reading college age textbooks in junior high and high school.” Melissa attended a special school for gifted and talented students, explaining, “Yes, I went to an all honors high school—and yes, all AP courses… I was accepted at a selective enrollment gt (gifted and talented school), a test-in school.”

Rachel explained the range of advanced classes in which she participated: “I took honors global studies and civ, honors geometry, honors algebra two with trig, then AP US history, AP language and comp for English, and then another one.” A few of those who reported having the opportunity to participate in these types of advanced classes reported some challenges they encountered in one or two of the classes, as Susan explained, “Yes, in high school, I took mostly honors classes and some AP classes in English, Social Studies and Spanish, but I was challenged in Math.”

Of the 11 (27%) participants who were not able to enroll in advanced and accelerated classes, most attended smaller independent high schools that did not offer either honors or advanced classes. These participants often explained, however, that they were able to participate in other advanced learning opportunities in their smaller high schools. Asher’s response was representative, as he explained,

I had an amazing teacher who gave me opportunities to participate in projects. One of my mentors was a science teacher and she also gave me advanced opportunities. She gave me so many chances for advanced work. She let me apply science to the real world and to me, that was beautiful. She gave me the confidence to know that I could go beyond and do extra work. There were so many people that believed in me and gave me advanced courses and advanced independent projects. They gave me time to write short stories. They let me explore my strengths and interests. They nurtured me—they gave me a fluid opportunity. Many public schools are like conveyer belts, but my school was like the elevator in Charlie and the Chocolate Factory.

### Extracurricular Activities

An interesting finding in this study was the high number of students who participated in sports, clubs, or extracurricular activities, as 36 (90%) of the participants reported various types of involvement in these experiences. These types of extracurricular experiences seemed to suggest a preference for individual versus team-based sports, and their choice also seemed to reflect more interest in STEM, as opposed to the humanities clubs. In order of frequency, they reported participating in the following sports: swimming, track and field, tennis, and cross country. In order of frequency, they reported participation in robotics, computer and coding clubs. As Molly explained:

Robotics team—I loved that as I struggled a lot socially growing up but in robotics, I liked having a team. It made me feel good about myself… I was part of the women’s science club. I founded a girls who code club. I was a math tutor. I am a STEM nerd.

Dylan also had broad representative experiences with extracurricular activities, noting:

Tennis, documentary film, senator for senior year, mock trial, band, and choir. Every year we make a film for a competition, there’s a topic, we made one about education declining as, there are funding problems in my town. I did it for two years.

Jackson also had a representative response,

Yes. I played soccer for 10 years but dropped it in high school. I play guitar a lot, I played piano, music was my hobby. I did some stuff in school. Outside of school, I did a lot of volunteer stuff for the town. We helped serve in community outreach stuff. That is how I got my first job at a farmer’s market—I set up tents and moved stuff, etc. I worked at a farm as well.

In addition to Molly, six participants indicated that they had founded various extracurricular activities, suggesting that for some in this study, educators enabled them to participate in activities in which they had strong interests. Melissa explained, “Yes. I was in Shakespeare club—I started that in school but I did a lot of stuff outside of school, I did improv, I did singing lessons, I played the flute, I did band for one year only.”

In summary, the vast majority of our participants reported positive and indeed transformative extracurricular activities that changed over time. Their discussion of these opportunities suggests that extracurricular activities were beneficial to developing their interests, their social understanding and their ability to work with others, as well as emerging and first-time leadership opportunities, as several started or led clubs and activities in areas of interest, most often in smaller schools.

### Residential Programs

When asked about their participation in residential or camp programs, half of the participants (50%) explained that they had been involved in a residential program away from home, either in middle and high school or both, usually in the summer, but also occasionally during school breaks in the academic year. They reported attending overnight theme camps, such as coding or filmmaking camps, sports camps, swim camps, and boy scout programs, which were beneficial to their preparation to live with others and improve their social and communication skills.

Avery explained, “Yes, through middle school and high school I would go to a summer camp every year and sleep at a campsite, and a winter retreat every year.” Walter reported that he had attended a 2-week filmmaking program at a college during his junior year of high school, adding, “It helped me to prepare for college.” Several other participants agreed, explaining, generally without being asked, that these programs were very helpful for their subsequent college success, enabling them to learn to live on their own, learn to socialize with others, and helping their communication skills.

As Edgar explained, the summer residential program he attended “…really helped as it helped my ability to communicate with others and helped me to understand how to explain what I need to explain to others, especially in my area of science.” Sasha also attended a residential program, explaining that,

Yes. I did stay away at a preparatory program in art overnight—the trick is to have each student make a schedule and understand what happens when you don’t follow your schedule. Without a schedule, you are lost—students who are ASD, have trouble finishing homework and also have trouble understanding the consequences of not doing their work. Without parents, students who go to college are often lost in scheduling time to do work.

These participants attended residential programs before leaving for college and believed that these experiences were beneficial and contributed to their success both in high school and college by exposing them to social situations that required new competences, such as understanding scheduling and time management and improving their communication skills.

### Understanding Their Academic Strengths and Weaknesses

Participants were asked whether they had learned about their academic strengths and weaknesses during their school experiences and their responses varied. Less than half (43%) said that they had an understanding about these while they were in elementary, middle, or high school, but that they had different perceptions about it at various times in their lives. As Penny explained,

I knew early, I think as early as 5 years old. It was innate. I came more into the identity and community aspect in college, but in high school, it was just me and it didn’t affect me, it was just an identity. No one helped me figure out strengths and needs.

Chris also believed he understood his profile by the time he was in high school.

Yes, I knew by high school or middle school. I understand my strengths and weaknesses. I was fully aware of my profile. By high school, I understood it a lot better and could control the weaknesses. Rather than let them go out of control, I handled them. For example, I knew that I was not good in social groups, so I would plan how to handle these before they happened. I had a few friends from clubs and elementary school and that experience made me better at dealing with social stress and anxiety.

Some of the participants, however, were confused by dual messages relating to their diagnosis as having ASD and academic strengths. As Walter explained,

However, I’ve been told different definitions of what it [ASD] means, and I think some are better than others. I always knew that I was smart. I didn’t feel smart in high school –sometimes I have a voice in my ear that tells me I am not smart.

Some of the participants had interesting perspectives about regarding their identification as ASD, trying to see it as a strength, as Kevin explained:

I did understand, it meant that autism meant I could go faster, but I needed time to pick the direction. When I have an idea of what I'm supposed to do, a point of interaction like seeing a math problem written out or using a computer program, I can usually figure it out quickly. But when I’m on my own it takes longer. The autism had to be explained to me. The special needs stuff was offered to me, like extra time on exams, or having a period of time to work on assignments as opposed to free periods, stuff to help me stay on track, make it to the work quickly, pick directions to work quickly.

Another group of 16 (40%), reported that they did not understand their ASD and strengths in high school. For these participants, the struggle to understand their disabilities existed in high school and continued into college, during the period in which they were interviewed. The majority of these respondents did explain that they retrospectively gained an understanding of the areas in which they excelled as well as their areas of weakness subsequent to their high school graduation, but they still had doubts.

Other participants did not. As Emily explained, “I don’t feel like I understand my academic strengths. I know I am really good at organization and planning ahead. I want to be a marketing data analyst.” Jackson also was able to discuss some of his strengths and weaknesses, reflecting on high school and his current situation:

I am not sure—I knew my academic strengths. I get a bit excessive at times. I overwork and freak out about things, especially big tests. Socially, I had huge weaknesses and I don’t put myself out there. I guess I appear to be distant and rude. I seem uncaring—my roommate is unhappy with me.

Most participants who reported that they did not understand their ASD in high school explained that they believed they had quirky personalities, and many simply explained that neither their parents nor teachers helped them to understand their dual diagnosis.

In summary, the majority of the participants believed that they had gained understanding of the intersection of their strengths and weaknesses in their elementary and secondary years. For example, Edgar reported that he had always been considered a precocious child, with intense interests and focus. He was identified as having ASD when he was five years old. He explained that he was labeled academically advanced and was able to skip two grades in mathematics in middle school. He is currently completing his first year at an extremely competitive university, majoring in meteorology, and acknowledges his close relationship with his family and his ability to pursue his interests as one of the primary reasons that he has been academically successful. Edgar had many opportunities to learn how to succeed in college from earlier experiences. For example, he attended a summer overnight preparatory program before starting college, as well as summer camps in meteorology for several years in another state. He also attended an astronomy camp based on his interests in another state. These residential experiences helped him learn how to communicate and live with others, as well as how to learn challenging content in all areas, but especially in his areas of intense interest, including science, meteorology, and technology.

### Necessary Academic and Meta-cognitive Skills and Strategies

When asked if they could go back to high school and learn skills to improve their academic performance and make their academic life in college easier, responses varied broadly. The most frequent response related to time management, as 6 of 40 respondents (15%) said they needed to understand how to handle their own time and schedule better. These students also discussed the need to better understand how to apply executive function skills and useful meta-cognitive strategies to their academic work, as well as how to control and organize independent scheduling of their time. Caleb explained,

I don’t know, but I think more long-term projects that would help with time management for me. Assigning different types of work—if there was a way they could acknowledge and understand different types of transition issues and they could help with scheduling.

Evan concurred, suggesting that,

the school should have taught me how to manage time better. In high school, we had no free time so I did not know how to make it better. School and college are two very different things. They should know that there is a difference between high school and college.

Some participants believed that they learned some of these skills at the summer programs they attended, as well as at their high schools, but the most frequent responses from this group included references to time management skill training, chunking out work over time, and making specific effort to get to know their teachers and professors. The majority of students, however, when asked about learning opportunities for meta-cognition could not clearly state the variety of meta-cognitive skills or executive function skills learned in high school that helped them succeed in college. They often discussed using these skills in other responses, and indeed may have used skills such as reflection about what worked well academically, awareness of strengths and weaknesses, knowledge of study and compensatory strategies, but could not explicitly explain their use in high school when asked.

As Edgar explained, “High school was about volume and college is about reacting to challenge and high quality.” In high school, Edgar learned to advocate for his own academic needs and hone his time management skills, adding that

I chunk my time out carefully and make sure that if I know I am going to need ten hours to do something, I plan to spend two hours every day on that task and then give it five days.

In summary, a small number of participants explained their use of meta-cognitive skills and self-regulatory skills, but most were limited to time management and an understanding of their strengths and weaknesses in learning. A few others were, however, able to explain their integration of executive function, defined primarily as actions that enable students to manage their time, focus on work that needs to be completed, and engage in multiple tasks to successfully achieve academic success.

## Discussion

Three themes emerged in this study that guide this discussion. Each of these was present across our in-depth data analysis of the transcripts of our 40 participants.

### The Importance of Advanced Content, Enriched Learning Opportunities, Interest and Choice

The participants in this study acknowledged the importance of several experiences that contributed to their academic success. For example, their interviews consistently addressed the positive consequences of their ability to succeed in advanced classes, as well as pursue their unique interests, and complete projects that they chose. Having time to pursue their academic interests was discussed by most participants, particularly those who attended smaller high schools. Participants discussed teachers who enabled and encouraged them to pursue advanced courses as well as advanced independent projects, based on interests. They also discussed the role of teachers who gave them the confidence to take academic risks, explaining that time to pursue advanced work in their areas of interest was important to them. The role of interests and choice appears to be very important to this population, as Asher explained, “When I am into something—I never stop.” Some previous research (Reis et al., 2014; Baum et al., 2014; Foley Nicpon et al., 2011; Wang & Neihart, 2014) has suggested that effective teaching for 2e students should focus on fostering students’ academic interests and strengths and this study strongly supports this view for 2e/ASD students. It appears clear that the use of advanced content, interest-based enrichment and strength-based strategies enhanced the academic success of these young people (Reis et al., 1997; Baum et al., 2014). Participants also explained that when interests guided their academic work, they were more motivated and engaged to gain advanced skills. Accordingly, the use of interest assessment (Reis et al., 2014) could benefit educational opportunities for this population both in advanced content acquisition, success in high school, and subsequent selection of college majors and careers.

The importance of interest, choice and advanced learning opportunities was also reflected in participants’ discussion of the value of extracurricular activities that they selected. Almost all, 90%, of the participants reported their involvement in various interest-based extracurricular experiences, reporting their participation in activities such as athletics, robotics, computer and coding clubs. Ten of the participants, without being specifically asked, discussed founding a club or activity or leading one of the clubs in their high schools. Matthew, for example, explained that he had started the video club which at one point had 60 members. Susan also played a leadership role, explaining that she was the director of the radio club.

Many of the students initially started in specific activities, then stopped and tried other clubs or teams. The role of choice appeared to be an important motivator in creating the enjoyment of these activities. Penny explained, “I was the editor for the school newspaper, I headed a literature club and GSA, but I dropped out of both. No sports at all.” Some research has suggested the importance of addressing students’ strengths and interests (Baum et al., 2014; Foley Nicpon et al., 2011), but in particular, research by Reis et al., (1997) found that 2e students who excelled in extracurricular activities were also subsequently able to be recognized for academic talents.

### Ability to Connect, Compensate, and Counteract

The second theme that emerged from the data was when faced with challenges, difficulties, or adverse conditions, rather than give up, these participants used their intelligence to find a reasonable path, figure out how to cope, compensate, or take a new direction. Connections with their support systems were also very important to them. Sometimes they switched classes or schools, at other times, they relied on their ability to reason and make a smart decision. Because of their ability to deeply consider their own actions, they often reflected on the challenges they faced, reasoned out what would have worked better, and charted a new direction. The participants encountered a challenge, such a social problem or a difficult class, and instead of giving up, most took corrective actions. They reached out to their support systems, tried to understand what they had done wrong in a social interaction, considered why they did poorly on an exam, tried to understand why they had difficulty with a class, arranged for additional help with disability services, made an appointment with a professor if they did not understand something, even when it was hard for them, and took corrective actions. For example if they needed to request a new roommate or a single room, they learned to do this. Many gave credit to their caring and emerging support systems over time, including their parents, teachers, and friends from their past and present, for their academic success.

### Intersections of Academic Talents and ASD

The third theme that emerged from these data was the intersection of academic talents and ASD. All of the participants were successful academically in competitive colleges, but as would be expected, some were more focused, directed, and exceled, while others worked diligently to achieve high grades but could not be classified as excelling. However, all were on the path to graduate, or had recently graduated, often with strong GPAs and career plans. Due to their academic talents, some acknowledged their college classes were not as challenging as they might have been, eliminating one major stressor in their lives. Their understanding of their ASD and patterns of strengths and weaknesses increased as they completed high school and attended college and as they learned to use their academic strengths to address these challenges.

### Implications

Educators and parents who support 2e students should focus on having their talents recognized, as it was important for these participants to have been identified as gifted or as having academic talents, enabling their inclusion in more advanced content classes and strength-based educational opportunities. As several participants were identified after completing comprehensive testing for the ASD, special education teachers and administrators should try to focus on students’ strengths and talents, as well as their disabilities, as a result of testing for special education services. As has been advocated in previous research (Reis et al., 2014), inclusion of talent development experiences and advanced learning opportunities made an important difference in the future academic success of this group, as did these participants’ identification as 2e.

Educators and parents of 2e students should advocate for the inclusion of talent development opportunities, especially extracurricular activities as goals for 2e/ASD students. The IEP or Sect. 504 plan can also include engaging and interest-based extracurricular activities such as those in which these students participated, including science fair, invention convention, or debate club. These extracurricular interest areas can help address 2e/ASD students’ social skills and leadership capabilities, using their interests and talents. Parents and educators should also consider the ways they can support these students’ social development, academic interests, and future college experiences by exposing them to residential programs that will help them to develop socially which will aid in their meta-cognitive development and understanding of how they learn. For these participants, residential programs attended during middle and high school were essential contributions to their subsequent academic success.

Students who are identified as 2e/ASD should be given opportunities to develop their interests and talents in both school and extracurricular activities. They should also have the opportunity to learn about their profiles of both strengths and weaknesses and understand the implications of their dual identification. They should be given information about why they should participate in advanced classes, extra-curricular activities, and residential programs, and they should have the opportunity to ask questions about why these different activities can be beneficial, even if they are uncomfortable initially.

### Limitations

Limitations exist in this study. The participants volunteered to be interviewed, and as a group, may be more motivated and socially able than other 2e/ASD students. All interview data were self-reported and therefore may include inaccuracies. However, we are confident about the participants identification as ASD, as our primary method for recruitment was by contacting centers for students with disabilities in competitive universities. Each of these centers require extensive documentation of their disability to qualify for disability services, and all participants in this study qualified and received those services. The majority of our participants were male and white, but we focused on recruiting young women and students with ASD from diverse populations as well, with almost a quarter of our participants being women, and 16% of all participants were from diverse cultural backgrounds.

Other limitations include the volume of data that results in time-consuming analysis and interpretation. In this study, over 300 pages of interview data were generated, and our analysis was guided by these and the creation of a code book, data matrices, and frequency tables. Research quality in qualitative studies is dependent on the skills of the researchers and can be influenced by the researchers’ personal biases, which is why all researchers participated in conducting interviews, coding data, and identifying findings and themes. Also, all researchers had previous and extensive experience in qualitative research methods.

Despite these limitations, our study has strengths. It includes the largest group of interviews of students identified as 2e/ASD reported in the professional literature. 2e/ASD students display similar yet unique profiles of talents and deficits, but some common experiences in high school may contribute to their academic success and positive transition to college. Understanding these experiences may enable other students with this profile to succeed in high school. Notably, this study can help to inform educators and parents and students who are 2e/ASD about which high school academic and social experiences were the most beneficial for this population of individuals who are academically talented with ASD. Their first-person interviews were a significant strength of the current study.

## Conclusions and Future Directions

Individuals who are 2e, especially those with ASD, have dreams, hopes, and goals to complete high school, attend and complete competitive colleges, and for some, attend graduate schools. They hope to work in an area of personal interest and choice, contribute to society, and live productive and happy lives, although they understand that their ASD will cause challenges. The importance of challenge, choice, and identification as having talents as well as disabilities was clear in our study, which suggests how little we know about this population, members of whom are at risk for not being identified, challenged, and supported. Young adults with talents and ASD need to have clear paths to academic success, guided by academic and extra-curricular activities that support their talents, while simultaneously, addressing their disabilities. A need also exists to develop and evaluate academic opportunities, resources, and encouragement (Reis et al., 2014) that can guide both talent development and special education goals, especially among young adults who will attend competitive colleges. Further investigation of the experiences of 2e/ASD students who both succeed and struggle in high school and college is necessary to create research-based support programs and enhance services for this group of individuals.
